# BanglaVerb: A sentence-level dataset for transitivity classification in Bangla NLP

**DOI:** 10.1016/j.dib.2026.112605

**Published:** 2026-02-18

**Authors:** Zannatul Mawa Koli, Md. Jahidul Alam, Zakia Sultana, Aliza Ahmed Khan

**Affiliations:** Department of Computer Science and Engineering. Daffodil International University, Bangladesh

**Keywords:** Natural language processing, Bangla language, Verb classification, Transitivity, Lexical resources

## Abstract

This article presents BanglaVerb, a systematically curated and linguistically validated sentence-level dataset designed to support transitivity classification in Bangla. The dataset contains 3001 Bangla sentences, each centered on a single verb instance annotated as either transitive (1634) or intransitive (1367). It was developed to address the lack of verb-focused linguistic resources for Bangla, a morphologically rich but under-resourced language in the NLP domain. Sentences were collected from diverse public sources, standardized, and carefully cleaned to ensure textual integrity. Annotation combined rule-based pre-labeling with expert linguistic verification, resulting in a 92% majority-voting agreement among annotators, which reflects high labeling consistency and reliability. Beyond its annotation framework, the dataset provides detailed lexical and structural statistics, including vocabulary size, character length distributions, n-gram patterns, and frequency distributions that follow Zipf’s law, confirming its linguistic representativeness. Baseline experiments using multiple machine learning models demonstrate strong classification performance, indicating the dataset’s clarity and robustness. By bridging sentence-level structure with verb semantics, BanglaVerb offers a high-quality, openly accessible resource that can support a wide range of downstream applications, including lemmatization, morphological analysis, syntactic parsing, semantic role labeling, and the development of verb-aware language models for Bangla.

Specifications Table

**Table:** 

Subject	Computer Sciences
Specific subject area	Bangla Natural Language Processing (NLP); Sentence-level transitivity classification; Linguistic resource development; Morphological and syntactic annotation.
Type of data	Text Files (xlsx-formatted)
Data collection	The dataset was compiled from publicly accessible Bangla text sources, including online news portals, blogs, magazines, and public social media pages, collected during 2023–2024. Sentences containing a single dominant verb were selected, cleaned, and normalized into Unicode-compliant Bangla script. Transitivity labels were assigned through a rule-based pre-labeling step followed by manual validation using majority voting by native Bangla speakers. Data preprocessing and organization were performed using Python (v3.10) with Pandas and NumPy.
Data source location	Bangla text collected from publicly accessible sources, including online news portals, blogs, magazines, and public Facebook pages.
Data accessibility	Repository name: Mendeley Data Data identification number: 10.17632/dczbv2tdn7.2 Direct URL to data: https://data.mendeley.com/datasets/dczbv2tdn7/2 Instructions for accessing these data: Open access; data can be freely downloaded from the repository without restriction.
Related research article	None

## 1 Value of the Data

• The dataset provides a comprehensive and well-structured collection of Bangla sentences containing both transitive and intransitive verbs. This resource is essential for building linguistically informed natural language processing (NLP) applications in Bangla, a low-resourced language with rich morphological variation.
• It addresses a critical resource gap in Bangla NLP by offering carefully annotated transitivity labels and linguistic attributes that support a range of downstream tasks such as lemmatization, part-of-speech tagging, morphological analysis, and semantic role labeling.
• The dataset enables researchers and developers to design and fine-tune language models, machine translation systems, and information extraction pipelines with a stronger grammatical understanding of verb usage. This contributes to improved contextual comprehension in applications like speech recognition, chatbot development, and text summarization.
• From a linguistic perspective, the dataset holds substantial value for computational morphology research, enabling detailed analysis of verb conjugation patterns, syntactic roles, and their variation across formal and informal contexts.
• By including a diverse range of verb forms, including regionally common and less standardized usages, the dataset can help reduce model bias and enhance inclusivity in Artificial Intelligent (AI) systems for Bangla speakers from different socio-linguistic backgrounds.
• Beyond NLP, this resource is also valuable in education, anthropology, and digital humanities, supporting curriculum design for Bangla grammar learning, language preservation efforts, and cultural archiving.
• Making the dataset openly accessible creates opportunities for collaborative research among linguists, computational linguists, and AI developers, fostering advancements in Bangla language technologies and encouraging the development of localized AI solutions.

## 2 Background

Verbs play a central role in the grammatical structure and semantic interpretation of sentences. In Bangla, a linguistically rich language with complex inflectional and derivational morphology, verbs are essential for expressing tense, aspect, voice, and argument structure. These characteristics make verb processing particularly challenging for natural language processing (NLP) applications, including part-of-speech tagging, lemmatization, semantic role labeling, and machine translation. Despite Bangla being one of the most widely spoken languages in the world and extensively used in digital communication, dedicated computational resources focusing on verb usage remain scarce.

In recent years, the Bangla NLP community has made notable progress in developing specialized datasets for downstream tasks. Large-scale corpora have been created for handwritten character recognition [1], tense classification [2], sentence transformation [3], sarcasm detection [4], lemmatization [5], and relation extraction [6]. These resources have advanced Bangla NLP by enabling more accurate and context-sensitive language models. However, most of these datasets are sentence- or token-based without explicit verb-centric annotation, despite verbs being key to understanding syntactic structures and semantic relations.

This gap becomes even clearer when compared to high-resource languages such as English [7,8], Chinese [9,10], or French [11,12], where verb-focused datasets, annotated with tense, aspect, valency, or transitivity, have significantly contributed to linguistic modeling, machine translation, and semantic understanding. In contrast, the lack of a structured and curated verb resource for Bangla limits the development and evaluation of models capable of capturing its grammatical complexity [13]. Specifically, the classification of transitive and intransitive verbs, an essential linguistic property, remains underrepresented in existing resources. To address this gap, we introduce BanglaVerb, a systematically curated sentence-level dataset consisting of 3001 Bangla sentences, each centered around a single verb instance categorized as either transitive or intransitive. The dataset was carefully compiled, cleaned, and statistically analyzed to support both linguistic research and NLP model development.

The primary objective of this work is to provide a structured and annotated resource that captures verb transitivity at the sentence level, unlike existing datasets that focus primarily on token- or sentence-level classification without explicit grammatical labeling. This dataset can support a variety of downstream applications, including lemmatization, machine translation, dependency parsing, and semantic role labeling, while also enabling fine-grained analysis of verb usage and distribution in Bangla.

This study presents the first openly accessible Bangla dataset with explicit transitivity annotation, accompanied by comprehensive descriptive statistics to facilitate linguistic exploration and machine learning. By integrating seamlessly with modern NLP workflows, it provides a strong foundation for the development of verb-aware models for Bangla and opens pathways for future expansions, including tense, aspect, and conjugation tagging, strengthening the low-resource language landscape and fostering deeper research in Bangla NLP.

## 3 Data Description

The Indian subcontinent is home to remarkable linguistic diversity, with Bangla occupying a particularly prominent position. Spoken primarily in Bangladesh and the Indian state of West Bengal, Bangla ranks as the seventh most spoken language in the world, with approximately 272.8 million native speakers [14]. Despite its widespread use, Bangla poses considerable challenges for natural language processing (NLP) due to the limited availability of linguistically rich resources that adequately capture its syntactic and stylistic variation. In particular, the absence of annotated corpora makes it difficult to model key grammatical phenomena such as the distinction between transitive and intransitive sentence structures. To address this gap, we introduce BanglaVerb, a carefully curated dataset consisting of 3001 Bangla sentences designed to support syntactic and semantic modeling of verb transitivity. Sentences were collected from publicly accessible Bangla text sources, including online news portals, magazines, blogs, and public social media pages. This diverse collection ensures broad coverage of contemporary language use and stylistic variation.

The dataset comprises 1634 transitive and 1367 intransitive sentences, achieving a near-balanced class distribution. This balance is crucial for training and evaluating models that can accurately capture and generalize the structural differences between transitive and intransitive constructions. Each sentence is annotated with transitivity labels and accompanied by contextual information in both Bangla and English, enabling fine-grained linguistic and computational analysis. The dataset was compiled with particular attention to lexical variety, morphological structure, and syntactic diversity, making it a valuable resource for a range of NLP tasks, including morphological analysis, syntactic parsing, and transitivity classification. Table 1 summarizes the key components and attributes of the BanglaVerb dataset, highlighting its organization and annotation framework.

**Table 1:** Key components and attribute descriptions of the Bangla Verb dataset.

Attribute	Description and Possible Values	Example
Sentence	Includes Bangla example sentences demonstrating the transitivity category of every verb.	Transitive: Transitive: Intransitive: Intransitive:
Verb	Denotes the transitivity category of the sentence. Every entry is designated as either: • Transitive — verbs that need a direct object. • Intransitive — verbs that do not need a direct object.	Transitive / Intransitive
Sentence_en	Offers the English rendition of the related Bangla sentence to aid in cross-lingual analysis and multilingual NLP applications.	Transitive: I read books. Transitive: You open the door. Intransitive: He runs. Intransitive: I sleep.

**Fig. 1:**
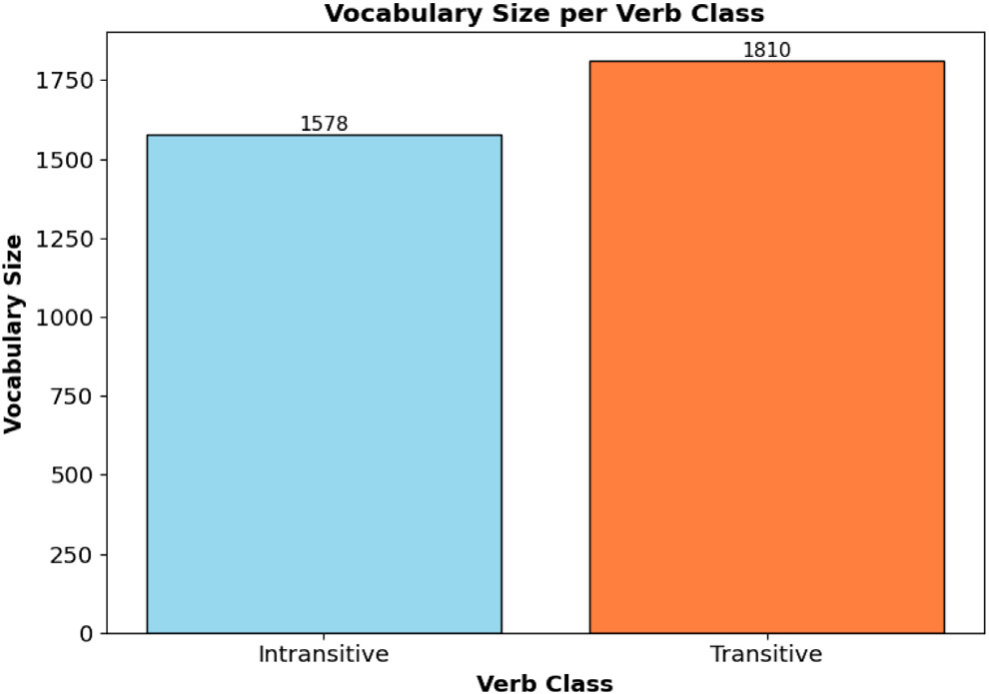
Vocabulary size per verb class.

**Fig. 2:**
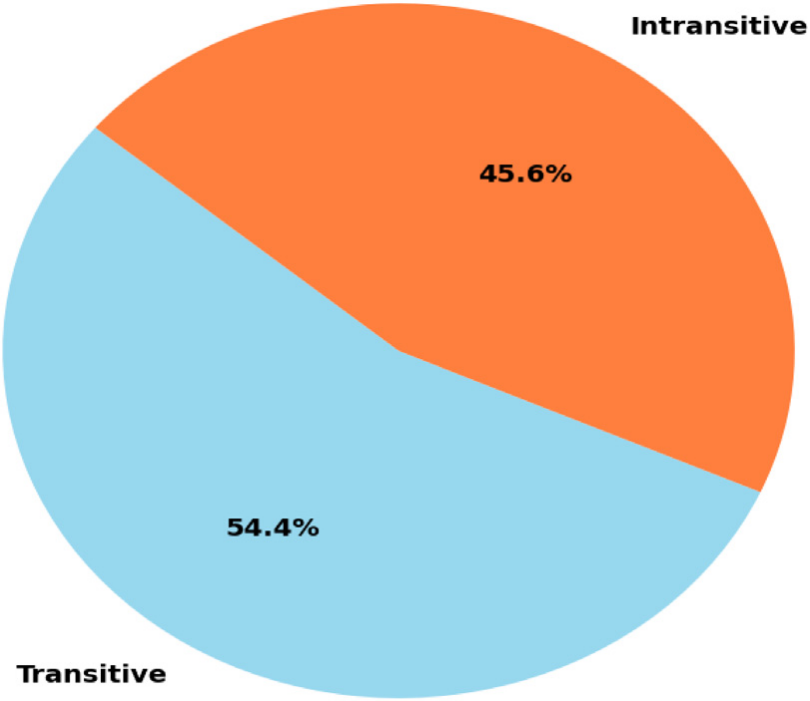
Class distribution of verbs.

The vocabulary size across classes reflects the dataset’s lexical richness. As shown in Fig. 1, the transitive sentence category contains 1810 distinct tokens, whereas the intransitive category includes 1578 unique tokens, indicating approximately 14.7 % higher lexical diversity for transitive sentences. This pattern aligns with the syntactic nature of Bangla, where transitive verbs typically occur with a broader range of objects and adjuncts. Fig. 2 further illustrates the class distribution, with transitive sentences accounting for 54.4 % of the total entries and intransitive sentences representing 45.6 %. This near-balanced distribution is essential for supporting robust and generalizable model performance across both verb types.

**Fig. 3:**
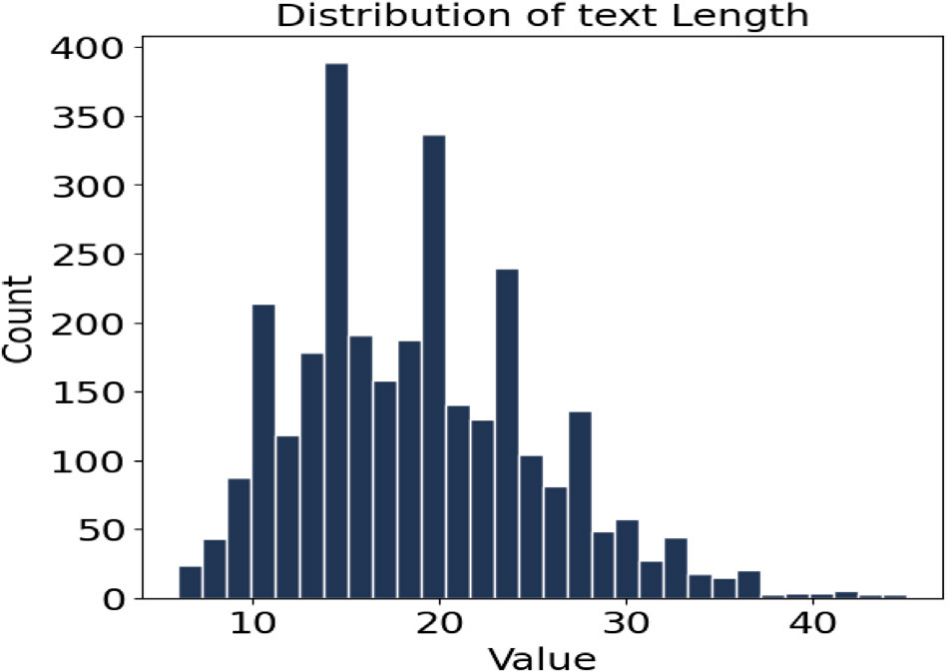
Character length distribution (overall).

Descriptive statistics on character length highlight the morphological complexity of the dataset. As summarized in Table 2, the average character length across all sentences is 18.77, with a standard deviation of 6.47, a minimum of 6 characters, and a maximum of 45. The first quartile (Q1) falls at 14 characters, the median (Q2) at 18, and the third quartile (Q3) at 23, indicating a right-skewed distribution. Fig. 3 further illustrates that approximately 67 % of all sentences fall within the 14–23-character range, reflecting a moderately complex lexical structure with a smaller proportion of longer, morphologically richer forms.

**Table 2:** Summary statistics of character length (overall).

Statistic	Value
Count	3001
Mean	18.77
Std. Dev.	6.47
Min	6.00
25 %	14.00
50 % (Median)	18.00
75 %	23.00
Max	45.00

**Fig. 4:**
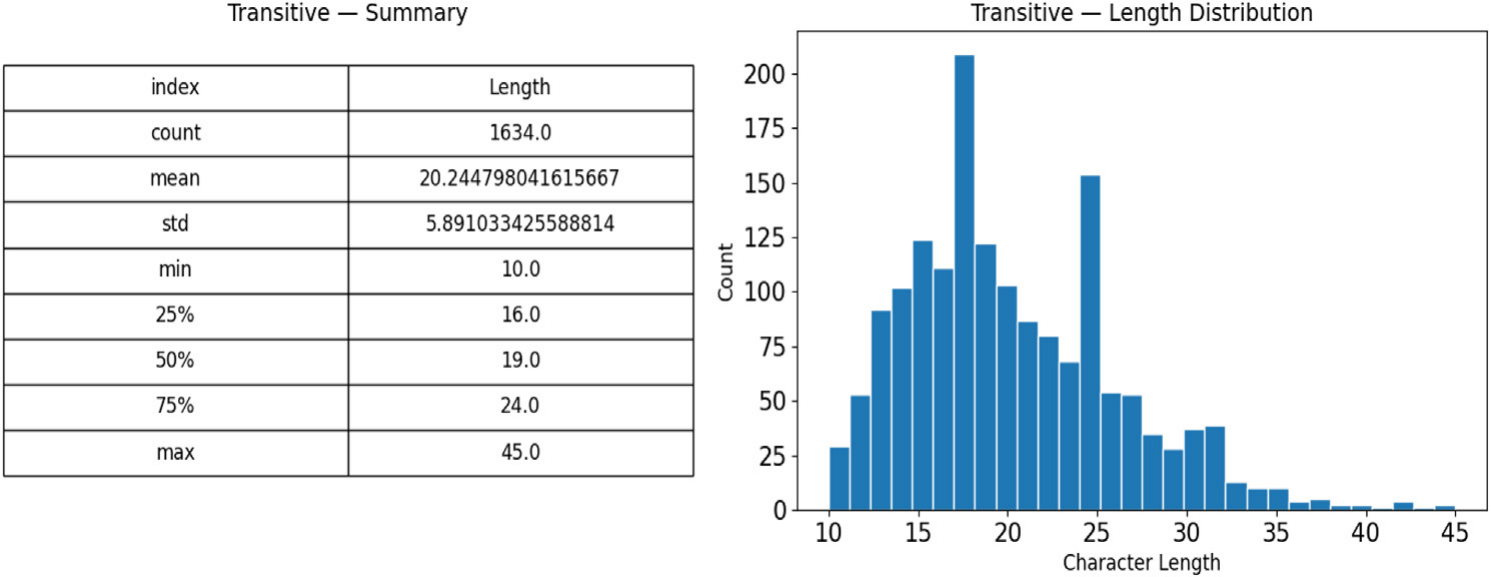
Transitive verb length summary and length distribution.

**Fig. 5:**
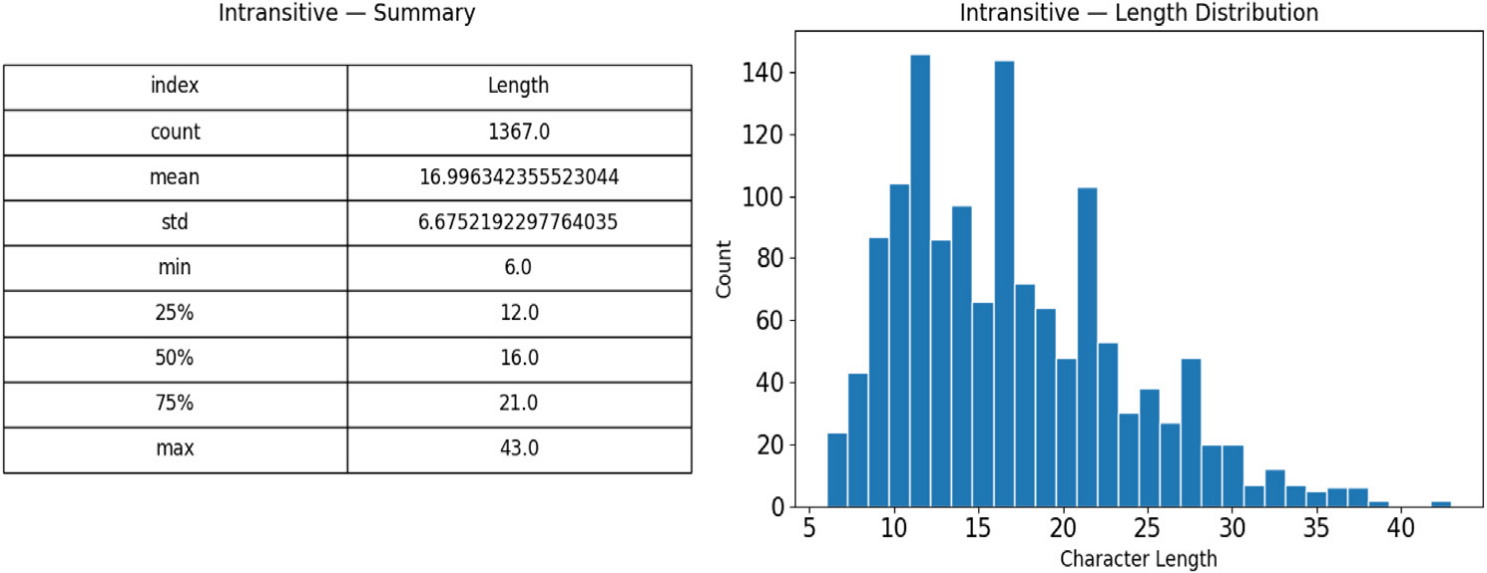
Intransitive verb length summary and length distribution.

A class-wise analysis reveals more nuanced structural variations within the dataset. As shown in Fig. 4, transitive sentences exhibit an average character length of 20.24, reaching a maximum of 45 characters, with Q1 = 15, Q2 = 19, and Q3 = 25. In contrast, Fig. 5 indicates that intransitive sentences have a lower average length of 17.00 and a maximum of 40 characters, with Q1 = 13, Q2 = 17, and Q3 = 21. The transitive distribution peaks between 16 and 24 characters, with 22.6 % of entries exceeding 25 characters, whereas the intransitive distribution is more concentrated in the 10–20-character range, with only 9.8 % surpassing 25 characters. This disparity highlights the greater morphological diversity found in transitive constructions compared to their intransitive counterparts.

**Fig. 6:**
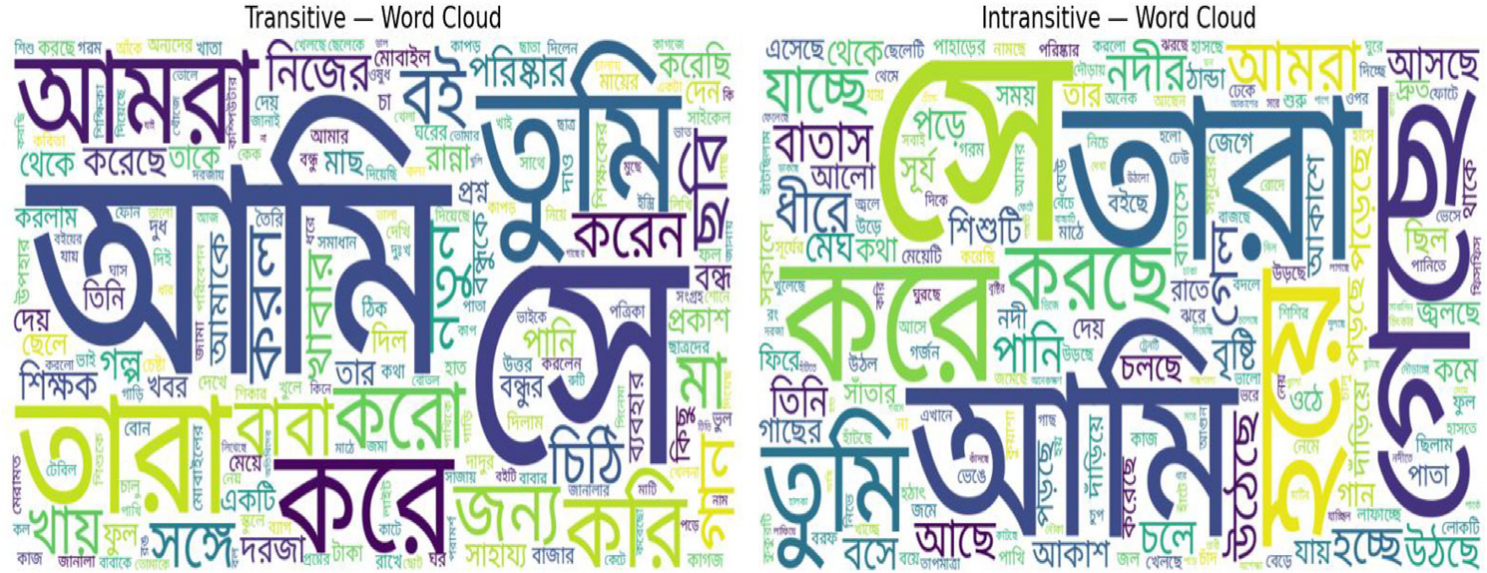
Word clouds of example sentences (Bangla).

**Fig. 7:**
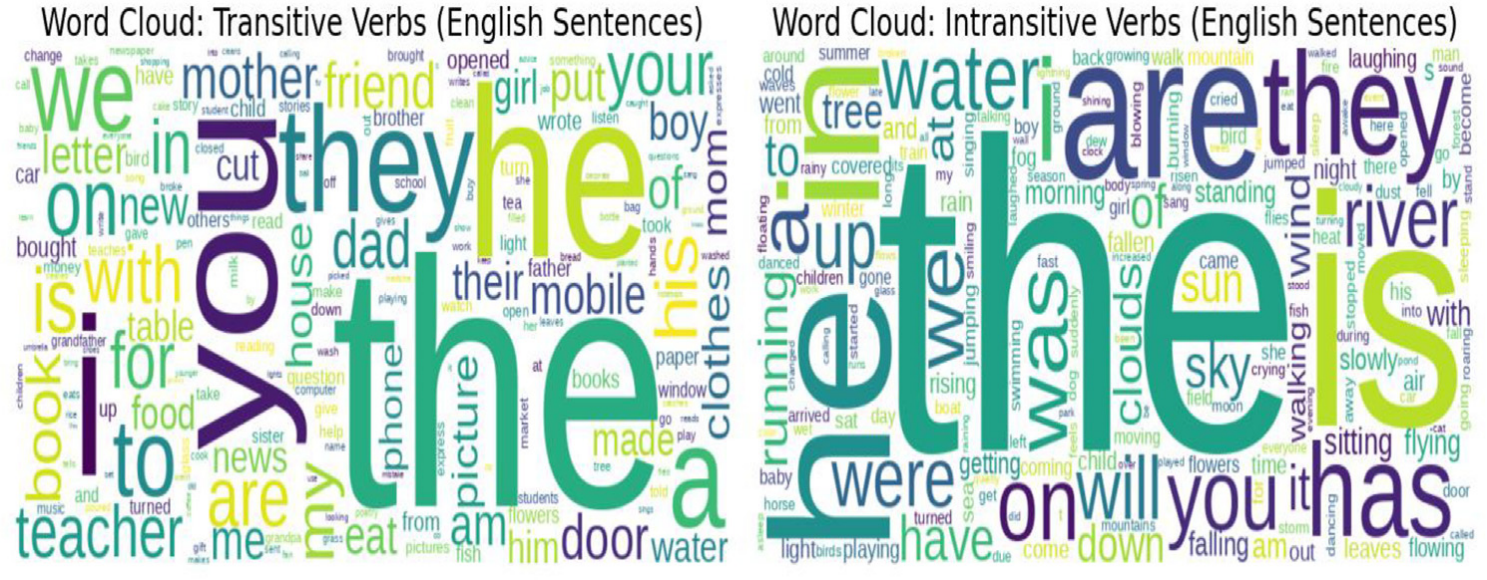
Word clouds of example sentences (English).

Lexical patterns derived from the example sentences provide valuable insights into how transitive and intransitive constructions are used in natural contexts. As shown in Fig. 6, the Bangla word clouds highlight frequent lexical items that cluster distinctly around each verb type. In transitive sentences, high-frequency tokens such as  (412 occurrences),  (301),  (329), and  (225) reflect common subject–verb–object structures typical of everyday communication. In contrast, intransitive sentences prominently feature tokens like (95), (37),  (23), and  (26), which often convey actions or states that do not require a direct object. Fig. 7 illustrates a similar trend in the English translations. Transitive sentences frequently co-occur with words such as “you” (316), “the” (1704), “they” (331), and “he” (535), indicating prototypical subject–object constructions. In contrast, intransitive sentences lean toward descriptive and environmental terms, including “is” (461), “river” (37), “sky” (33), and “clouds” (26), which align with events or conditions expressed independently of an object. These lexical patterns underscore clear contextual and structural distinctions between the two transitivity types.

This contrast between the two transitivity classes highlights not only differences in lexical density but also clear contextual divergence in sentence structure. Supporting this observation, the n-gram analysis provides a structured view of the most frequent syntactic patterns. As presented in Table 3, unigrams such as  (506),  (412), and  (301) dominate Bangla sentences, while bigrams like  (25) and trigrams such as  (5) reveal common collocational structures. In English (Table 4), similar patterns emerge, with “in the” (131), “on the” (85), and “he is” (76) among the most frequent bigrams, and trigrams like “in the sky” (15) and “the river water” (11) indicating recurring thematic constructions. Taken together, these lexical and structural patterns offer a richer linguistic perspective, illustrating how transitivity influences surrounding lexical choices and syntactic configurations in both Bangla and English.

**Table 3:** Top unigrams, bigrams, and trigrams (Bangla).

**Table 4:** Top unigrams, bigrams, and trigrams (English).

Rank	Unigram	Frequency	Bigram	Frequency	Trigram	Frequency
1	the	1704	in the	131	in the sky	15
2	he	535	on the	85	in the morning	11
3	is	461	he is	76	in the field	11
4	i	399	they are	63	the river water	11
5	they	331	i am	51	turn on the	10
6	you	316	the teacher	46	the child is	10
7	a	255	the door	41	the girl is	10
8	are	191	the river	39	the clouds are	9
9	in	182	of the	37	flying in the	8
10	we	172	to the	33	turned on the	8

Collectively, these analyses demonstrate that the BanglaVerb dataset embodies a rich lexical variety, a well-balanced class distribution, and comprehensive morphological and contextual characteristics. The statistical patterns observed in character length, vocabulary size, and lexical frequency provide strong evidence of its linguistic robustness. These properties make the dataset a well-defined and reliable resource, suitable for a wide range of downstream applications, including morphological analysis, syntactic parsing, and semantic modeling in Bangla NLP.

## 4 Experimental Design, Materials and Methods

**Fig. 8:**
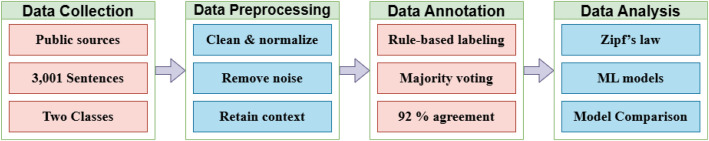
Workflow of the dataset development process, including data collection, preprocessing, annotation, and analysis stages.

The experimental design of this study was systematically developed to ensure both high data quality and strong linguistic validity. As illustrated in Fig. 8, the workflow consisted of four structured stages. First, 3001 Bangla sentences containing verb instances were collected from publicly accessible Bangla text sources, including online news portals, blogs, magazines, and public social media pages, to ensure broad linguistic and stylistic coverage. In the second stage, the data underwent comprehensive cleaning and normalization to remove inconsistencies and standardize textual representation. The third stage focused on annotation, combining rule-based pre-labeling with expert linguistic verification to ensure accuracy and consistency. Finally, in the fourth stage, statistical evaluations and model-based analyses were carried out to assess the overall quality and reliability of the dataset, confirming its robustness for a wide range of downstream NLP applications.

### 4.1 Data preprocessing

A comprehensive pre-processing pipeline was applied before annotation and analysis to maintain consistency and textual integrity throughout the dataset Raw verb entries were gathered from publicly accessible Bangla text sources, including blogs, magazines, online news portals, and public social media pages, ensuring diverse linguistic coverage. These entries were then carefully cleaned to remove duplicates and non-lexical noise such as incomplete tokens or stray symbols.

Each verb was normalized into Unicode-compliant Bangla script to resolve encoding issues and standardize text representation. Non-essential punctuation was stripped away, but meaningful markers that carry grammatical or contextual significance were retained. To ensure uniform spelling and structure, orthographic variations were standardized using a combination of manual correction rules and morphological heuristics, while preserving authentic language forms. Unlike many pre-processing pipelines, stopwords were deliberately retained, as they play an important role in contextual interpretation, especially in verb-centered sentence structures. This multi-step process produced a clean, standardized dataset of 3001 sentences entries with transitivity labels and aligned usage contexts, providing a solid foundation for both linguistic analysis and NLP model training.

### 4.2 Data annotation

To ensure high linguistic accuracy and consistency, the annotation of the Bangla Verb dataset was carried out through a carefully structured semi-automated and manual process. In the first stage, each of the 3001 sentences entries was automatically pre-labeled as either transitive or intransitive using a set of curated grammatical rules derived from standard Bangla syntax. These initial labels served as a baseline for expert validation.

In the second stage, the pre-labeled entries were reviewed by a pool of five native Bangla speakers with linguistic expertise. Each annotator examined three key aspects: (1) the lemma form, to ensure the verb’s root representation was accurate; (2) the transitivity classification, to determine whether the verb required a direct object; and (3) the sentence context, to verify whether the classification aligned with actual language use.

Every entry was evaluated by at least three annotators. In cases where opinions diverged, the final label was determined through a majority voting mechanism. Any ambiguous or borderline cases were escalated to a senior linguistic expert for resolution. This process resulted in 1634 transitive and 1367 intransitive sentences, achieving a 92 % agreement under the majority-voting procedure, calculated as the percentage of sentences receiving the same transitivity label across annotators, indicating strong consistency and reliability in the dataset’s labeling (Table 5).

**Table 5:** Example of annotation process with majority voting for verb classification.

### 4.3 Data analysis

**Fig. 9:**
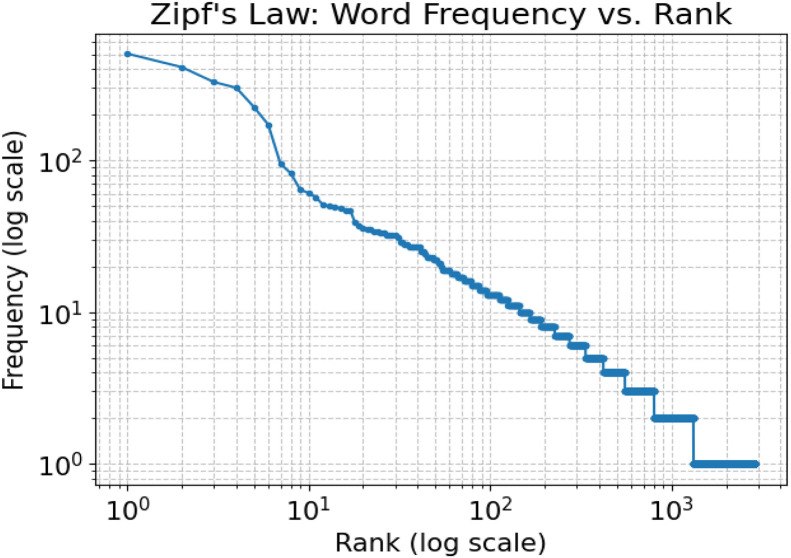
Zipf’s Law applied to the Bangla Verb Dataset: Word frequency vs. rank (log–log scale).

To evaluate the lexical and statistical characteristics of the Bangla Verb dataset, we performed a detailed analysis that combined frequency distribution modeling with supervised learning experiments. Our first step focused on examining whether the lexical distribution of the dataset reflects typical natural language behavior. To do this, we applied Zipf’s law, which posits that the frequency of a word is inversely proportional to its rank within a corpus. The resulting log–log plot, illustrated in Fig. 9, clearly shows a classic power-law distribution. A small set of high-frequency verbs, such as  (“to be”),  (“to do”),  (“to go”), and  (“to eat”), occupies the top ranks, creating a steep initial slope. As the rank increases, the curve flattens into a more linear region that captures mid-frequency verbs like  (“to take”) and  (“to write”). The tail of the curve stretches across hundreds of low-frequency verbs, including those with morphological variations or regional specificity. This distribution mirrors the expected long-tail behavior observed in natural languages, highlighting how a few core verbs dominate usage while a broad spectrum of less common verbs contributes to linguistic richness and diversity.

To further assess the quality and reliability of the dataset, we conducted a binary classification task to distinguish between transitive and intransitive verbs. Six widely used machine learning algorithms were employed: Logistic Regression, Support Vector Machine (SVM), Decision Tree, Random Forest, XGBoost, and CatBoost. A stratified split of 601 annotated samples was used to train and evaluate all models under identical experimental settings, ensuring fair comparison across architectures. As shown in Table 6, the best overall performance was achieved by XGBoost, reaching an accuracy of 88.85 %, with precision, recall, and F1-score all at 0.89. CatBoost followed closely with 87.52 % accuracy, while Logistic Regression, SVM, and Random Forest each attained 86.52 %. The Decision Tree model, used as a simple baseline, recorded an accuracy of 75.04 %. These results demonstrate that the dataset enables robust and stable classification performance across a range of algorithms, highlighting the clarity, consistency, and balanced structure of its transitivity annotations.

**Table 6:** Model performance on transitivity classification task.

Model	Accuracy	Precision	Recall	F1-Score
Logistic Regression	0.8652	0.87	0.86	0.86
Support Vector Machine (SVM)	0.8652	0.87	0.86	0.86
Decision Tree	0.7504	0.75	0.75	0.75
Random Forest	0.8652	0.87	0.86	0.86
XGBoost	**0.8885**	**0.89**	**0.89**	**0.89**
CatBoost	0.8752	0.88	0.87	0.87

**Fig. 10:**
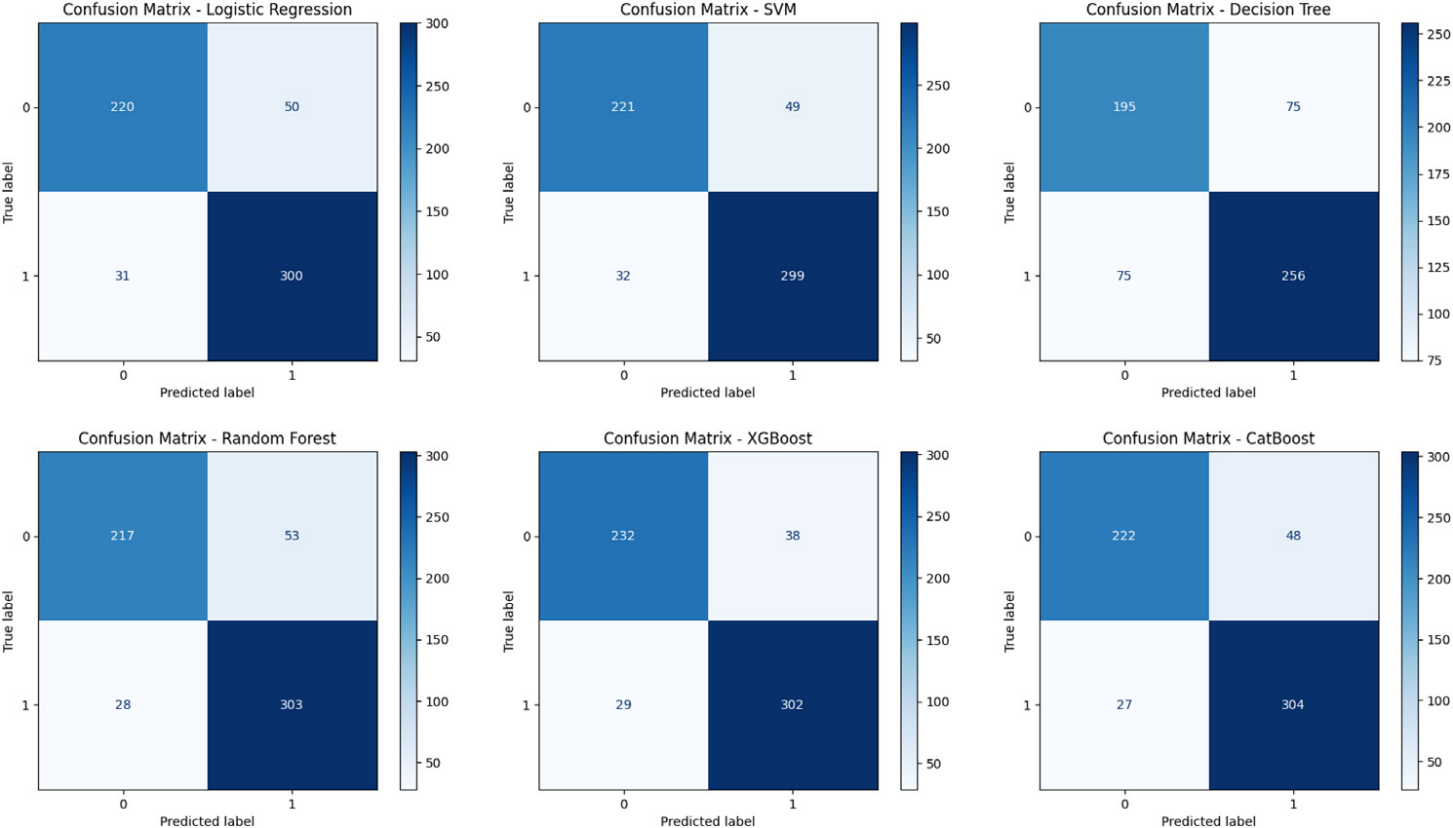
Confusion matrices for all models, showing class-wise prediction accuracy for transitive and intransitive verbs.

To gain deeper insights into model performance, confusion matrices were generated for each classifier (Fig. 10). Among the six algorithms, XGBoost produced the strongest results, correctly identifying 302 transitive and 232 intransitive verbs with minimal misclassifications. CatBoost performed comparably well, with 304 and 222 correct predictions for transitive and intransitive classes, respectively. Logistic Regression, SVM, and Random Forest exhibited slightly higher false negative rates but maintained similar overall accuracy trends. In contrast, the Decision Tree model displayed more dispersed errors, reinforcing its lower precision relative to the other methods. Importantly, most misclassifications were concentrated among verbs with ambiguous or context-dependent transitivity, pointing to specific linguistic areas where future dataset refinement could further enhance model performance.

**Fig. 11:**
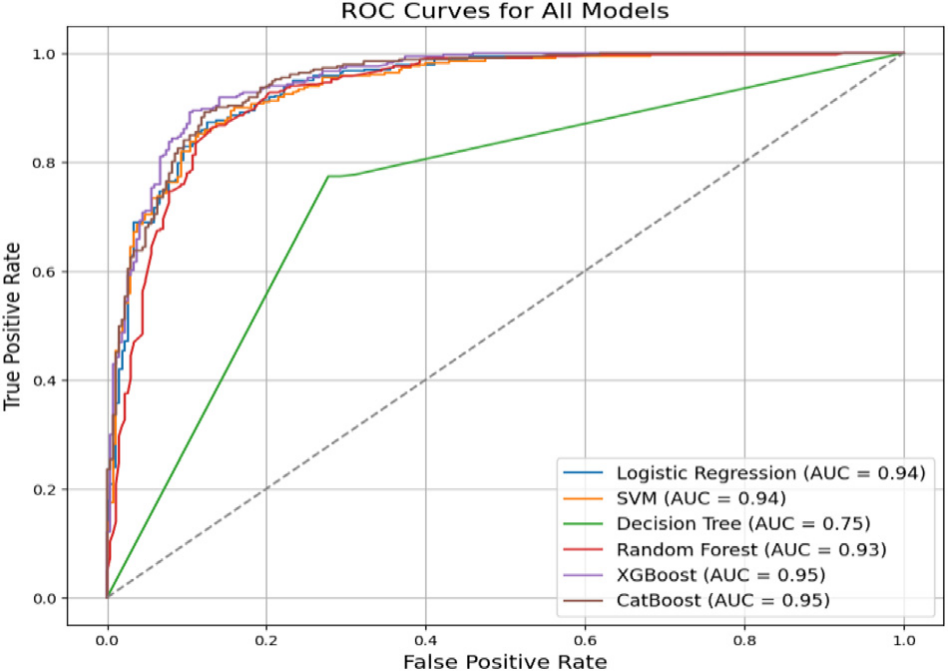
ROC curves for baseline models illustrating classification performance across thresholds.

Receiver Operating Characteristic (ROC) curves were generated to evaluate model performance across different classification thresholds. As illustrated in Fig. 11, XGBoost achieved the highest Area Under the Curve (AUC), closely followed by CatBoost, reflecting their strong discriminative capabilities. Logistic Regression, SVM, and Random Forest produced similar AUC values, slightly below the top performers, while the Decision Tree model exhibited the weakest curve. The consistently high AUC scores for the leading models highlight clear class separability, confirming that the dataset offers strong and reliable signals for accurately distinguishing between transitive and intransitive verbs.

These findings reveal that even relatively simple models can achieve strong performance on the verb transitivity classification task, reflecting the linguistic clarity and well-structured nature of the dataset. Moreover, the Zipfian distribution observed in the lexical analysis reinforces that the dataset closely mirrors natural language behavior. This combination of linguistic consistency and statistical robustness makes the resource highly suitable for a broad range of downstream NLP applications, including morphological analysis, language modeling, and verb-aware syntactic parsing.

## Limitations

While the BanglaVerb dataset provides a structured and linguistically rich resource, several limitations should be acknowledged. First, although the dataset’s size of 3001 sentences is suitable for baseline experimentation, it may not fully capture the full lexical and structural diversity of Bangla, particularly with respect to less common or dialectal forms. Second, the annotation process, despite achieving a high level of inter-annotator agreement, relied on manual expert validation and may still contain subtle subjective biases, especially in sentences where verb transitivity is ambiguous or context-dependent. Third, data collection primarily focused on standard and frequently used verb forms, which may result in underrepresentation of rare, archaic, or region-specific variations. Additionally, the example sentences were sourced from controlled linguistic environments rather than spontaneous spoken language or informal social media contexts, potentially limiting coverage of colloquial and conversational structures. Finally, as the dataset is monolingual at the verb level, with English included only as a gloss, its immediate applicability to cross-lingual NLP tasks may be constrained without further expansion.

## Ethics Statement

The authors confirm that they have read and fully comply with the ethical requirements for publication in Data in Brief. This study did not involve human participants, animal experiments, or the use of data obtained from social media platforms. All data were collected exclusively from publicly available linguistic resources and controlled text sources, with no personally identifiable information gathered or processed. Consequently, formal ethical approval and informed consent were not required for this research.

## CRediT authorship contribution statement

**Zannatul Mawa Koli:** Conceptualization, Methodology, Data curation, Formal analysis, Writing – original draft, Visualization, Project administration. **Md. Jahidul Alam:** Software, Visualization, Validation, Investigation, Writing – review & editing. **Zakia Sultana:** Data curation, Resources, Supervision, Writing – review & editing. **Aliza Ahmed Khan:** Data curation, Resources, Writing – review & editing.

## Data Availability

Mendeley DataBV-Class (Original data).
